# Chaperone-mediated autophagy promotes breast cancer angiogenesis via regulation of aerobic glycolysis

**DOI:** 10.1371/journal.pone.0281577

**Published:** 2023-03-13

**Authors:** Rui Chen, Peng Li, Yan Fu, Zongyao Wu, Lijun Xu, Junhua Wang, Sha Chen, Mingzhen Yang, Bingjie Peng, Yao Yang, Hongwei Zhang, Qi Han, Shuhui Li

**Affiliations:** 1 Tibetan Traditional Medical College, Lhasa, China; 2 Yantai Mountain Hospital, Yantai, Shandong, China; 3 General Hospital, Western Theater Command, Chengdu, Sichuan, China; 4 General Hospital of Tibet Area Military Command, Lhasa, China; 5 Army Medical University (Third Military Medical University), Chongqing, China; Medical College of Georgia at Augusta University, UNITED STATES

## Abstract

Evidence shows that chaperone-mediated autophagy (CMA) is involved in cancer cell pathogenesis and progression. However, the potential role of CMA in breast cancer angiogenesis remains unknown. We first manipulated CMA activity by knockdown and overexpressing of lysosome-associated membrane protein type 2A (LAMP2A) in MDA-MB-231, MDA-MB-436, T47D and MCF7 cells. We found that the tube formation, migration and proliferation abilities of human umbilical vein endothelial cells (HUVECs) were inhibited after cocultured with tumor-conditioned medium from breast cancer cells of LAMP2A knockdown. While the above changes were promoted after cocultured with tumor-conditioned medium from breast cancer cells of LAMP2A overexpression. Moreover, we found that CMA could promote VEGFA expression in breast cancer cells and in xenograft model through upregulating lactate production. Finally, we found that lactate regulation in breast cancer cells is hexokinase 2 (HK2) dependent, and knockdown of HK2 can significantly reduce the ability of CMA-mediated tube formation capacity of HUVECs. Collectively, these results indicate that CMA could promote breast cancer angiogenesis via regulation of HK2-dependent aerobic glycolysis, which may serve as an attractive target for breast cancer therapies.

## Introduction

Breast cancer is the most common cancer and the leading cause of cancer death in women worldwide, many factors contribute to initiation and progression of breast cancer [1, 2]. As angiogenesis is essential for tumor growth and metastasis, inhibiting tumor-associated angiogenesis has long been a promising target in limiting cancer progression [3, 4]. Vascular endothelial growth factor A (VEGFA) is a major player in angiogenesis among the VEGF family that promotes angiogenesis through binding and activating the tyrosine kinase receptor VEGFR2 [5]. Tumor cells always activate and initiate angiogenesis by releasing VEGFA to improve their microenvironment in many physiological and pathological conditions, such as hypoxia and high levels of lactate [3, 6]. Lactate, the obligatory product of aerobic glycolysis, plays an important role in angiogenesis, immune escape, cell migration and metastasis [7].

Cells undergo degradation of intracellular components by lysosomes, also known as autophagy, which is crucial for cellular homeostasis and adaptation to various forms of stress [8]. Of the three different kinds of autophagic pathways, chaperone-mediated autophagy (CMA) is unique for the selective degradation of damaged soluble cytosolic proteins by recognizing peptide sequence motif (KFERQ) via heat shock cognate protein of 70 kDa (HSC70) [9], then the targeted substrate binds to lysosomes through the lysosome-associated membrane protein type 2A (LAMP2A), key protein in the CMA [10, 11], and undergoes degradation. Recently, there has been growing interest in how CMA impacts cancer cell pathogenesis and progression. Blockage of CMA reduces tumor growth by reducing glycolytic flux and/or the accumulation of glycolytic intermediates in lung cancer cells and melanoma [12, 13]. CMA can also promote lung cancer cell survival through selective stabilization of the pro-survival protein, MCL1 [14]. Inhibition of LAMP2A also induces cell death by AKT1 and ROS pathway in breast cancer cells [15]. Evidence shows that key glycolytic enzymes, such as glyceraldehyde-3-phosphate dehydrogenase (GAPDH), and pyruvate kinase muscle isozyme (PKM), aldolase, and HIF1A are all identified as substrate of CMA [16, 17]. However, the link among CMA, glycolysis and breast cancer angiogenesis has not been explored. Thus, we hypothesized that CMA regulating breast cancer angiogenesis may be associated with aerobic glycolysis.

## Materials and methods

### Animal study

20 four-six-week-old female BALB/c nude mice in total were randomly divided into four groups, and each group had five mice (n = 5). Mice were held at the Experimental Animal Center of Army Medical University for one week before injection, and the health status and well-being of the mice were checked twice a day by the animal care staff. All animal studies were approved by the Ethics Committee of Army Medical University and all the procedures were performed according to the regulations made by the Experimental Animal Center of Army Medical University. Mice were injected with MDA-MB-436 cells (in 200 μl of serum-free RPMI-1640 medium at 1 × 10^7^ cells/mouse) after LAMP2A knockdown or overexpression at the right flank. When tumor volumes of negative and control groups reached about 150 mm^3^ (tumor size = length × width × width × 0.5), animals were sacrificed by CO_2_ euthanasia, and all efforts were made to minimize suffering, then the subcutaneous tumors were collected and used for Western blot.

### Cell culture

Human breast cell lines of MCF7, MDA-MB-231, T47D and MDA-MB-436 were obtained from the cell bank of the Committee on Type Culture Collection of the Chinese Academy of Sciences (CCTCC). Human Umbilical Vein Endothelial Cells (HUVECs) were from Cobioer Biosciences. MDA-MB-231, MDA-MB-436 cells and HUVECs were cultured in RPMI 1640 medium (Gibco), and T47D cells were cultured in Dulbecco’s modified Eagle’s medium (Gibco). MCF7 cells were cultured in MEM medium with 1% non-essential amino acids and 10 μg/ml insulin. All cells were supplemented with 10% fetal bovine serum (Gibco) and incubated in an atmosphere of 5% CO_2_ at 37°C.

### Antibodies and reagents

Antibodies were as follows: HK2 (2867) was from Cell Signaling Technology, LAMP2A (ab18528) and VEGFA (ab214424) were from Abcam, β-actin (BM0626) was from Boster, GAPDH (10494-1-AP) was from Proteintech. Opti-MEM I (31985–062) and 1% crystal violet (C0121) were from Beyotime, Lipofectamine 2000 (11668–027) and puromycin (A11138-03) were from Invitrogen. MTT (M5655) and DMSO (D4540) were from Sigma-Aldrich.

### Cell infection and transfection

The lentivirus suspension used for LAMP2A shRNA and overexpression vector was purchased from GenePharma. The target sequence for LAMP2A was 5’-GCAGTGCAGATGACGACAA-3’ (1283). MDA-MB-231, MCF7, T47D and MDA-MB-436 cells were infected with lentivirus-mediated LAMP2A shRNA or LAMP2A overexpression vectors, and then were stably selected by puromycin resistance. HK2 siRNA (GenePharma) sequence was 5’-GCAGAAGGUUGACCAGUAUTT-3’, and then cells were transfected with HK2 siRNA via lipofectamine 2000 according to the manufacturer’s instructions, and then cells were cultured for 48 h before being harvested.

### Lysosomal association of human recombinant his-GAPDH

Lysosomal association of human recombinant his-GAPDH was performed as described by our previous study [18]. Lysosome Enrichment Kit was from Pierce (89839). Briefly, intact lysosomes (100 μg protein) from breast cancer cells with different LAMP2A expression were isolated, incubated with purified recombinant protein of 50 μg his-GAPDH and 2 μg his-HSC70 (all prepared by our lab), and then collected by centrifugation, fractionated and immunoblotted with GAPDH antibody to detect CMA activity.

### Tumor-conditioned medium collection (TCM) and lactate detection

MCF7, MDA-MB-231, T47D and MDA-MB-436 with different expression of LAMP2A (1× 10^5^/well) were cultured in 6-well plates with 2 ml of the relative medium, incubated at 37°C and 5% CO_2_ for 24 h. And then tumor-conditioned medium was transferred to the 10 ml centrifuge tubes, centrifuged for 5 min at 1500 rpm. Supernatants can be stored at -80°C or used for relative experiment immediately. Lactate determinization was conducted according to the manufacture’s operation using Lactate Assay Kit (MBL International). Briefly, standard and samples were prepared, incubated with Reaction Mix and measured at OD 450nm.

### Tube formation assay of HUVECs

Tube formation assay of HUVECs was performed by pipetting 100 μl Matrigel (BD Biosciences, USA) into each well of 96-well plate, then incubated at 37°C for 30 min to form the ECM gel. HUVECs (2 × 10^4^) with 100 μl TCM from breast cancer cells after LAMP2A knockdown or overexpression were then added into each well and incubated for 12 h. Then cell was stained with 5 μM Calcein-AM for 30 min and then images were taken using fluorescence microscope (100 ×). The capillary tubes were quantified by counting the average numbers of completed tubule structures in five randomly selected fields.

### MTT assay

HUVECs (1×10^3^cells/well) were cultured with 100 μl TCM from breast cancer cells after LAMP2A knockdown or overexpression in 96-well plates. At day 3, cell medium was replaced by new breast cancer TCM. The cell numbers were counted by MTT assay according to the manufacturer’s instructions after an additional 0, 1, 2, 3, 4, 5, 6 and 7 days, respectively. Briefly, 20 μl of the MTT solution (5 mg/ml) was added to each well, and incubated for 5 h at 37°C. The solution was discarded, then the crystal was dissolved in 200 μl DMSO and detected at 490 nm.

### Colony formation assay

HUVECs were cocultured with 1 ml TCM from breast cancer cells after LAMP2A knockdown or overexpression, seeded (800 cells/well) onto 12-well plates and allowed to form colonies for two weeks. Then, the colonies were fixed with 4% paraformaldehyde and stained with 1% crystal violet. After rinsing, the colonies were counted under a microscope.

### Migration assay

For the migration assay, HUVECs were directly seeded in a Transwell chamber (Millipore, PIEP12R48) with 8-μm pores in the membranes and cultured for 8 h in a 24-well plate. HUVECs were seeded at 4 × 10^4^/well in the upper chamber with serum-free medium and 500 μl TCM from breast cancer cells after LAMP2A knockdown or overexpression was added to the lower chamber as a chemoattractant. Then, the cells in the upper chamber were wiped off and the cells on the lower surface were fixed and stained with 1% crystal violet, photographed and counted under a microscope.

### Wound-healing assay

HUVECs (1×10^5^ cells) were seeded in 24-well plates, after cells reached 100% confluency, a wound was created by a sterile 200 μl pipet tip. The detached cells were washed away by PBS and cells were incubated in 500 μl TCM from breast cancer cells after LAMP2A knockdown or overexpression for 36 h. Then the remaining wound was observed under a light microscope.

### Quantitative real-time PCR

Total RNA was extracted by using Trizol agent (Invitrogen, USA) according to manufacturer’s instruction. cDNA was synthesized by Bestar^™^ qPCR RT Kit (DBI^®^ Bioscience, Germany). Quantitative real-time PCR (qRT-PCR) was conducted on a Bio-Rad IQ5 Detection System with Bestar^®^SybrGreen qPCR mastermix (DBI^®^ Bioscience, Germany). Primers are listed as follows: VEGFA forward: CCCACTGAGGAGTCCAACATC, VEGFA reverse: CACCAACGTACACGCTCCA; HK2 forward: CAACTTCCGTGTGCTTTGGG, HK2 reverse: CAACGTCTCTGCCTTCCACT; FGF1 forward: 5’-CAGTGGATGGGACAAGGGAC-3’, FGF1 reverse: 5’-CCGTATAAAAGCCCGTCGGT-3’; EGF1 forward: 5’-TTGTCATGCTGCTCCTCCTG-3’, EGF1 reverse: 5’-GGTTGCATTGACCCATCTGC-3’; HGF1 forward: 5’-ACCCTGGTGTTTCACAAGCA-3’, HGF1 reverse: 5’-GCAAGAATTTGTGCCGGTGT-3’; Actin forward: AGCGAGCATCCCCCAAAGTT, Actin reverse: GGGCACGAAGGCTCATCATT.

### ELISA

VEGFA protein levels of TCM from breast cancer cells after LAMP2A knockdown or overexpression were measured by human VEGF ELISA Kit (NeoBioscience, EHC108.96, China) according to the manufacturer’s instructions. Briefly, sample was added to the microwell (100 μl/well), incubated at 36°C for 90 min, washed for 5 times, added with 100 μl of Biotin-Conjugate antibody, incubated at 36°C for 60 min, washed for 5 times, incubated with 100 μl of diluted Streptavidin-HRP at 36°C for 30 min in the dark, colored with TMB Substrate Solution for about 30 min, stopped with Stop Solution, and finally detected on a spectro-photometer at OD 450 nm. The values were then calculated from the standard curve.

### Extracellular acidification rate (ECAR) measurement

An XF-96 Extracellular Flux Analyzer (Seahorse Bioscience) was used for the extracellular acidification rate analysis. In brief, MDA-MB-231 cells were adhered to XF 96-well microplate (Seahorse Bioscience) at 8000 cells/well. Treatment were as follows: glucose (25 mM), oligomycin (1 μM), and 2-DG (50 mM).

### Western blotting

Western blot analysis was performed as described by our previous study [18]. Briefly, protein samples were harvested, subjected to 10% SDS-PAGE, transferred onto a polyvinylidene fluoride membrane, blocked and incubated with various primary antibodies at 4°C overnight, β-actin, LAMP2A, HK2 and VEGFA were used as primary antibodies. After incubation with secondary antibodies, the immunoreactive bands were detected by ECL kit (Millipore, USA).

### Statistical analysis

All values are presented as the mean ± S.E.M of three independent experiments. The results of the experiments were analyzed by unpaired Student’s t test, *p* < 0.05 was considered statistically significant. Data were analyzed using SPSS version 21 (IBM Corp. Ltd., USA).

## Results

### CMA promotes breast cancer angiogenesis *in vitro*

**Fig 1:**
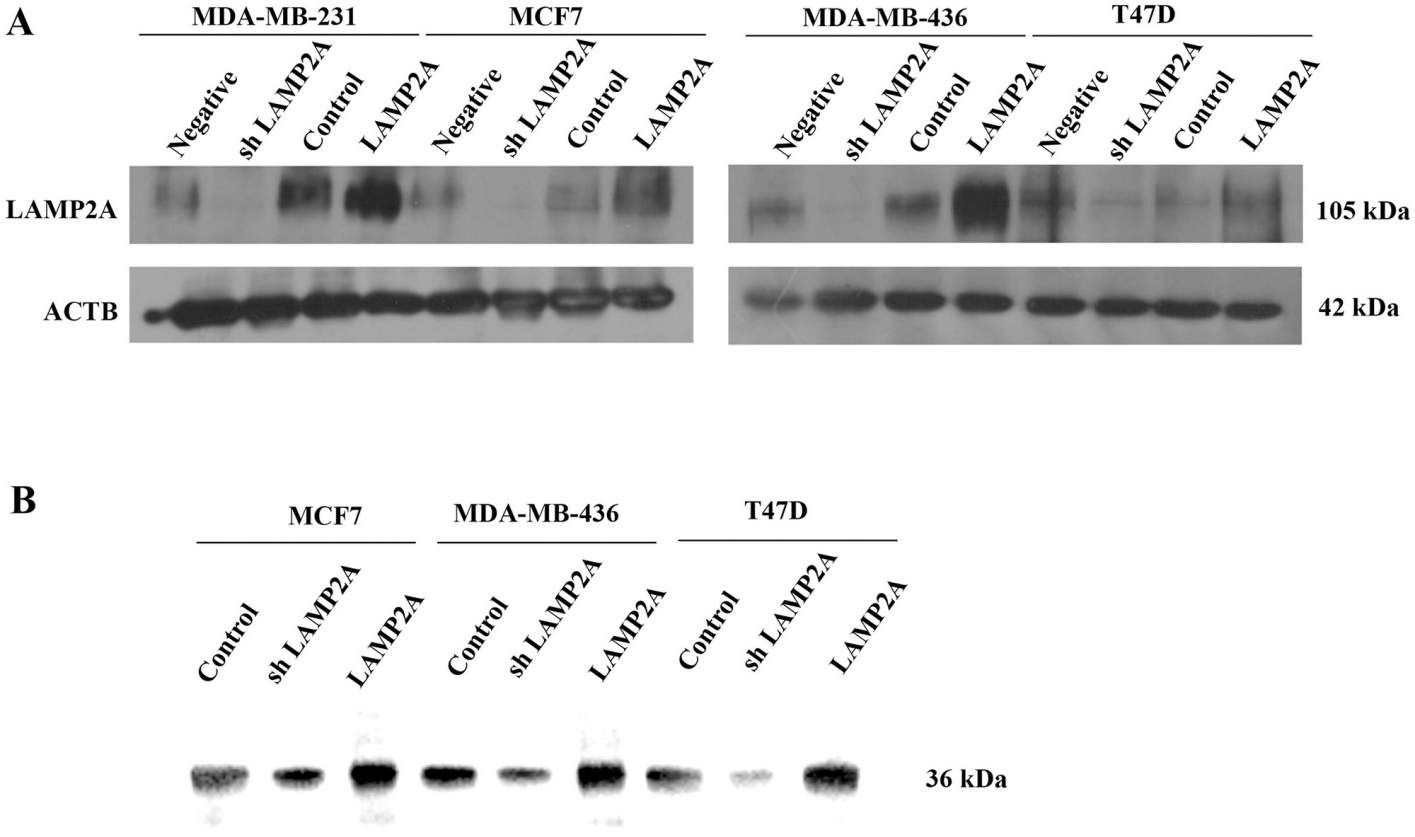
Establishment of breast cancer cell lines with different CMA activity. (A) Immunoblotting was used to detect the stable efficiency of lentivirus mediated inhibitory shRNAs or overexpression against LAMP2A in MDA-MB-231, MCF7, MDA-MB-436 and T47D cells. (B) Intact lysosomes from breast cancer cells with different LAMP2A expression was incubated with purified recombinant protein of GAPDH, and then collected, fractionated and immunoblotted with GAPDH antibody to detect CMA activity.

**Fig 2:**
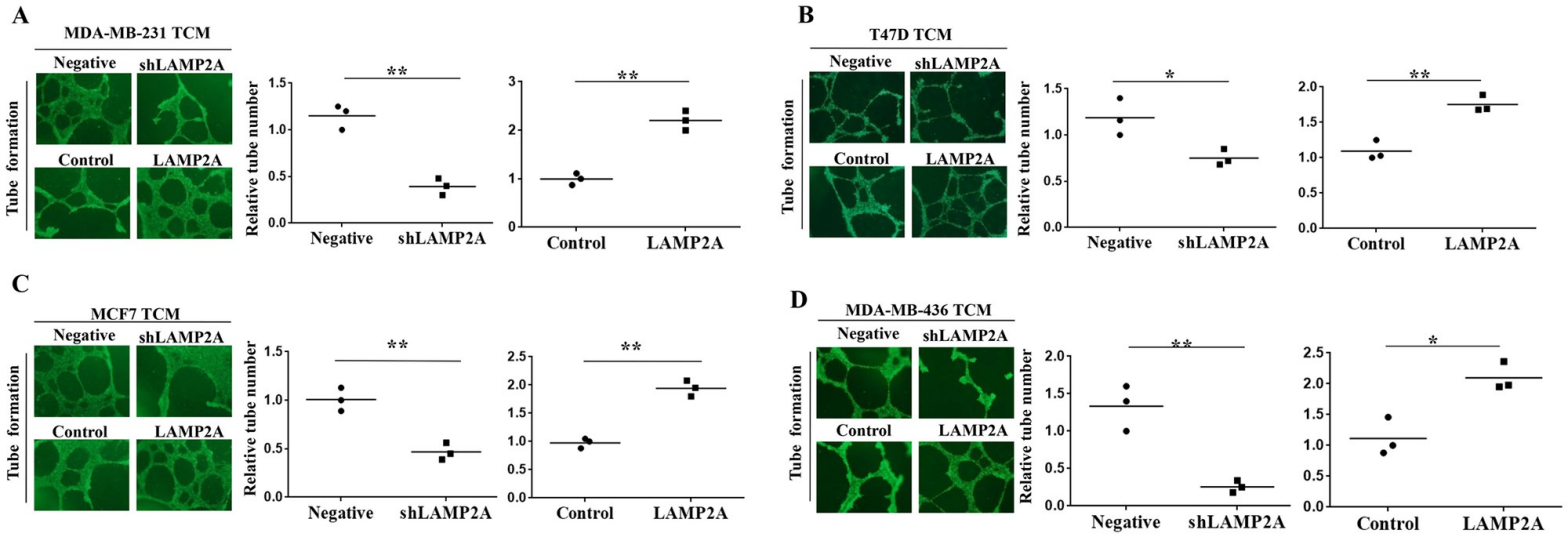
Chaperone-mediated autophagy promotes angiogenesis *in vitro*. (A) to (D) TCM was collected from different breast cancer cells after LAMP2A knockdown or overexpression, cocultured with HUVECs for 12 h, and then tubule numbers were counted via tube formation assay. *P < 0.05 and **P < 0.01. Data are representative of three independent experiments.

Our previous study [18] have successfully established two breast cancer lines with different LAMP2A expression and shown that CMA can promote breast cancer cell proliferation and metastasis *in vitro* and *in vivo*. In order to confirm the role of CMA in breast cancer angiogenesis, we first downregulated or upregulated CMA activity in three other breast cancer cell lines of MDA-MB-436, MCF7 and T47D by lentivirus-mediated LAMP2A knockdown and overexpression. The efficiency of LAMP2A overexpressing or silencing was verified by Western blotting (Fig 1A). CMA activity can be detected by a direct approach measuring the association of purified GAPDH with active intact lysosomes isolated in the presence of protease inhibitor from LAMP2A overexpressed or knockdown cells, and increased lysosomal association of GAPDH suggests higher CMA activity when compared with the control cells [15]. Lysosomal association of GAPDH was decreased in MDA-MB-436, MCF7 and T47D cells after LAMP2A knockdown, whereas lysosomal association of GAPDH was increased in these cells after LAMP2A overexpression (Fig 1B). We then explored the effect of CMA activity in breast cancer angiogenesis by tube formation assay *in vitro* [19]. HUVECs were cocultured with different TCM derived from breast cancer cells after LAMP2A knockdown or overexpression and cultivated on Matrigel to form tube. Fewer capillary tubes were formed in TCM from breast cancer cells of LAMP2A low expression, while more capillary tubes were observed in TCM from LAMP2A overexpression (Fig 2A–2D).

### CMA promotes proliferation and migration of HUVECs

**Fig 3:**
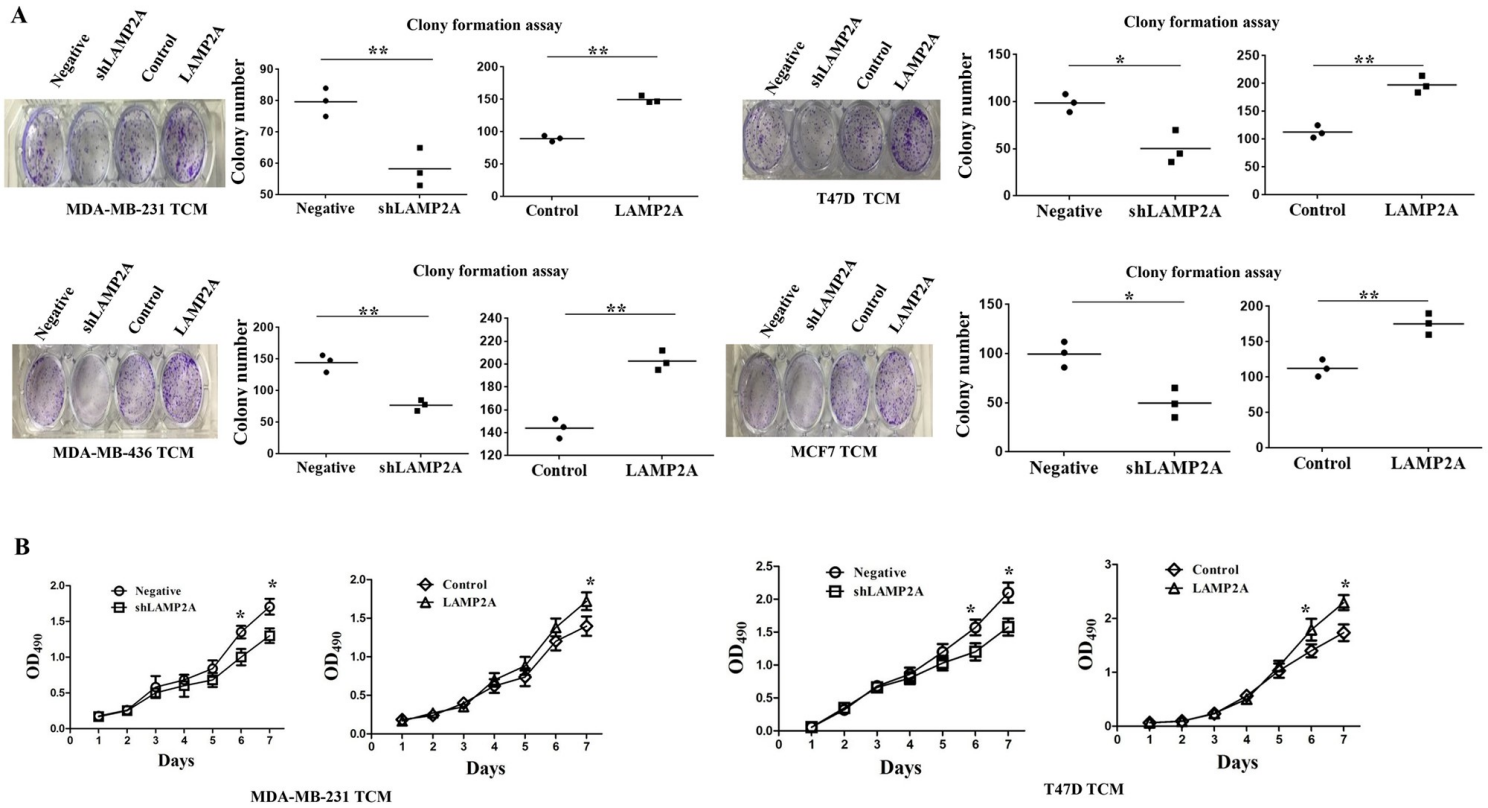
CMA promotes proliferation of HUVECs. (A) The proliferative capability of HUVECs was detected by colony formation assay. The cells were seeded onto 12-well plates, cocultured with TCM from breast cancer cells and allowed to form colonies for two weeks. (B) MTT assay was used to determine the proliferation rate of HUVECs cocultured with TCM at indicated timepoints. All values are representative of three different experiments; *P < 0.05 and **P < 0.01.

**Fig 4:**
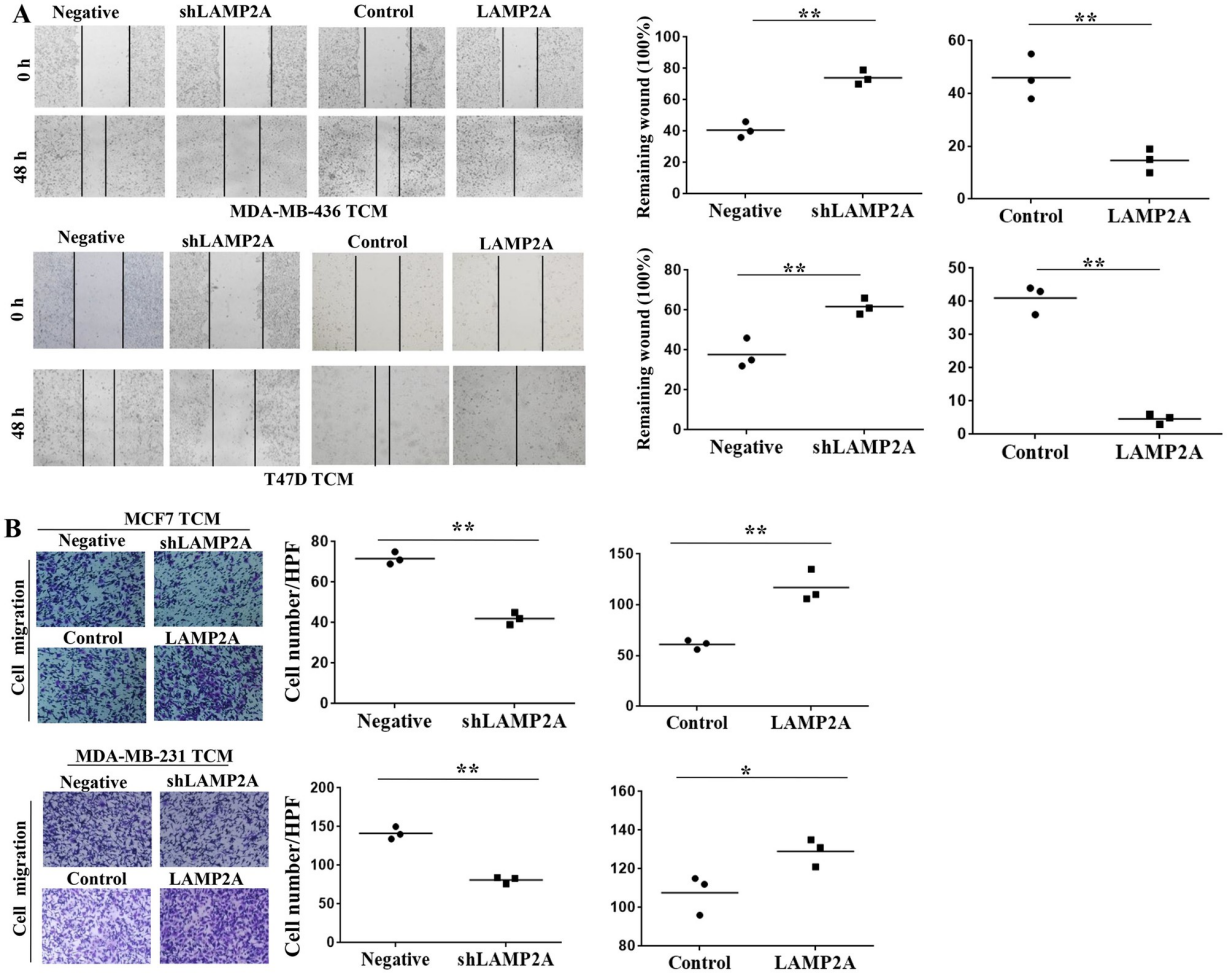
CMA promotes migration of HUVECs. (A) Wound-healing assay was used to detect the migration of HUVECs. After the wound scratch, the cells were cultured with TCM from MDA-MB-436 and T47D cells for 36 h and the size of the wound at indicated times was observed by microscope. (B) Transwell migration assay was used to determine the migration of HUVECs. HUVECs migrated to the lower chamber with TCM from MDA-MB-231 and MCF7 were counted under a microscope. All values are representative of three different experiments; *P < 0.05, **P < 0.01.

The tube formation capacity of HUVECs was associated with HUVEC proliferation and migration [20]. We first determined the effect of CMA activity on HUVEC proliferation by colony formation assay. We found that HUVEC proliferation rate was significantly decreased after cocultured with TCM from breast cancer cells of LAMP2A low expression, while HUVEC proliferation rate was significantly increased after cocultured with TCM from breast cancer cells of LAMP2A overexpression (Fig 3A). MTT assay further confirmed that TCM from breast cancer cells of LAMP2A knockdown inhibited HUVEC proliferation ability and TCM from LAMP2A overexpression promoted HUVEC proliferation ability (Fig 3B). As endothelial cell migration is also essential for angiogenesis [21], we then performed wound healing assay to detect the effect of CMA on HUVEC migration. We found that TCM from breast cancer cells of LAMP2A low expression downregulated HUVEC migration, while TCM from breast cancer cells of LAMP2A overexpression promoted HUVEC migration (Fig 4A). Transwell migration assay also demonstrated that HUVEC migration was decreased after cocultured with TCM from breast cancer cells of LAMP2A low expression and HUVEC migration was increased after cocultured with TCM from LAMP2A overexpression (Fig 4B).

### CMA promoted VEGFA expression in breast cancer cells

**Fig 5:**
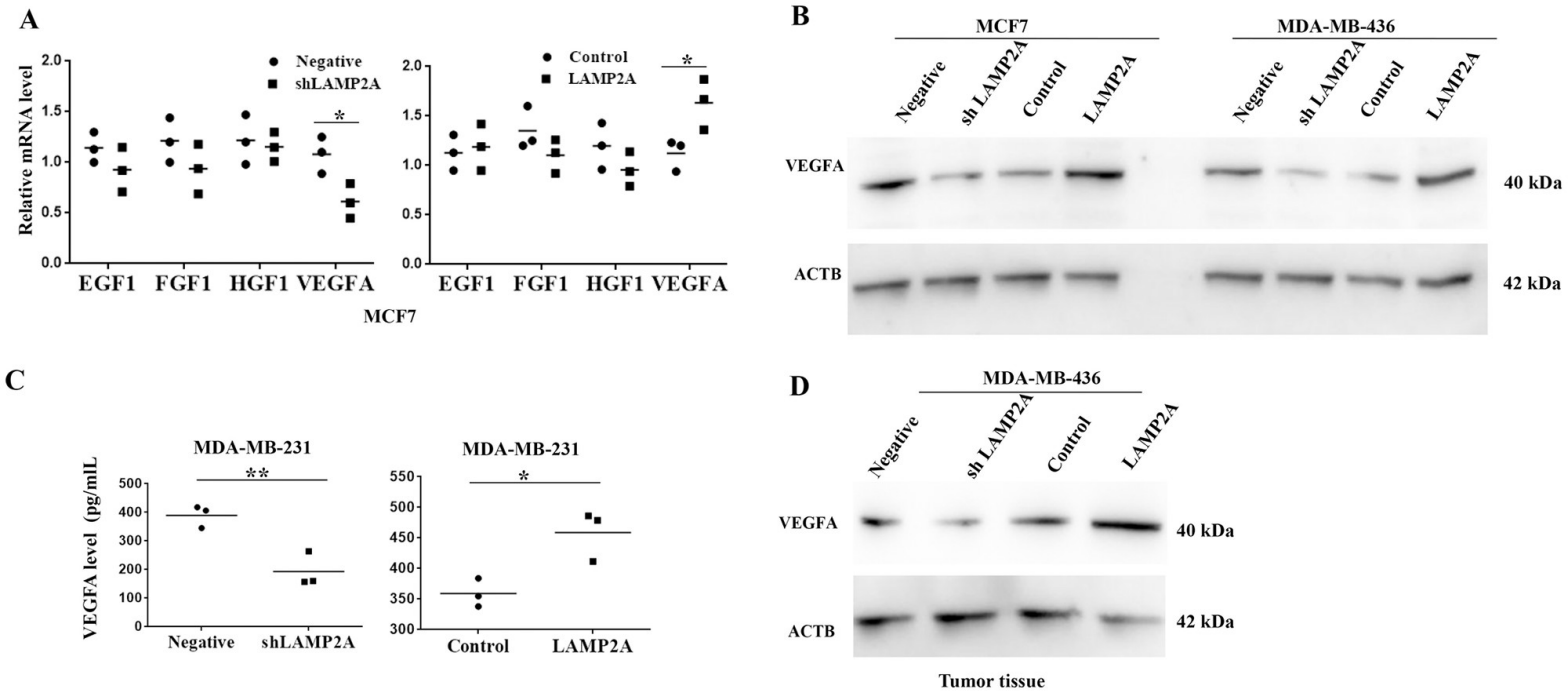
CMA promoted VEGFA expression of breast cancer cells. (A) VEGFA mRNA expression level was detected by real-time PCR in breast cancer cell line MCF7 after LAMP2A knockdown or overexpression. (B) The protein expression level of VEGFA was measured by Western blotting in MCF7 cells and MDA-MB-436 cells after LAMP2A knockdown or overexpression. (C) VEGFA level was detected by ELISA in breast cancer cell line MDA-MB-231 after LAMP2A knockdown or overexpression. (D) The protein expression level of VEGFA was measured by Western blotting in MDA-MB-436 xenograft tumor models after LAMP2A knockdown or overexpression. MDA-MB-436 cells with different LAMP2A expression, were subcutaneously injected into nude mice at 1×10^7^ cells/mouse. All values are representative of three different experiments; *P < 0.05, **P < 0.01.

Since CMA can regulate the process of angiogenesis, and VEGFA is the principal angiogenic cytokine [22], we then detected the expression and biological activity of VEGFA after manipulating CMA activity. We found that VEGFA mRNA level was significantly decreased after downregulation of LAMP2A in breast cancer cells, while LAMP2A overexpression could promote VEGFA mRNA expression level in breast cancer cells (Fig 5A). Then Western blotting further showed that LAMP2A knockdown led to decreased protein level of VEGFA in breast cancer cells, while LAMP2A overexpression promoted the VEGFA protein level in breast cancer cells (Fig 5B). ELISA assay also confirmed that VEGFA concentration was significantly downregulated in TCM from breast cancer cells of LAMP2A low expression and upregulated in TCM from breast cancer cells of LAMP2A overexpression (Fig 5C). Moreover, we used subcutaneous tumor model of xenograft in nude mice (tumor growth is shown in S1 Fig) to assess the VEGFA expression level by Western blotting, we found that LAMP2A knockdown significantly inhibited VEGFA expression in tumors from the shLAMP2A MDA-MB-436 implanted mice, compared with tumors from the negative MDA-MB-436 implanted mice, while LAMP2A overexpression significantly promoted VEGFA expression in tumors from the LAMP2A MDA-MB-436 implanted mice, compared with tumors from the control MDA-MB-436 implanted mice (Fig 5D).

### CMA promoted breast cancer angiogenesis through regulation of HK2-mediated aerobic glycolysis

**Fig 6:**
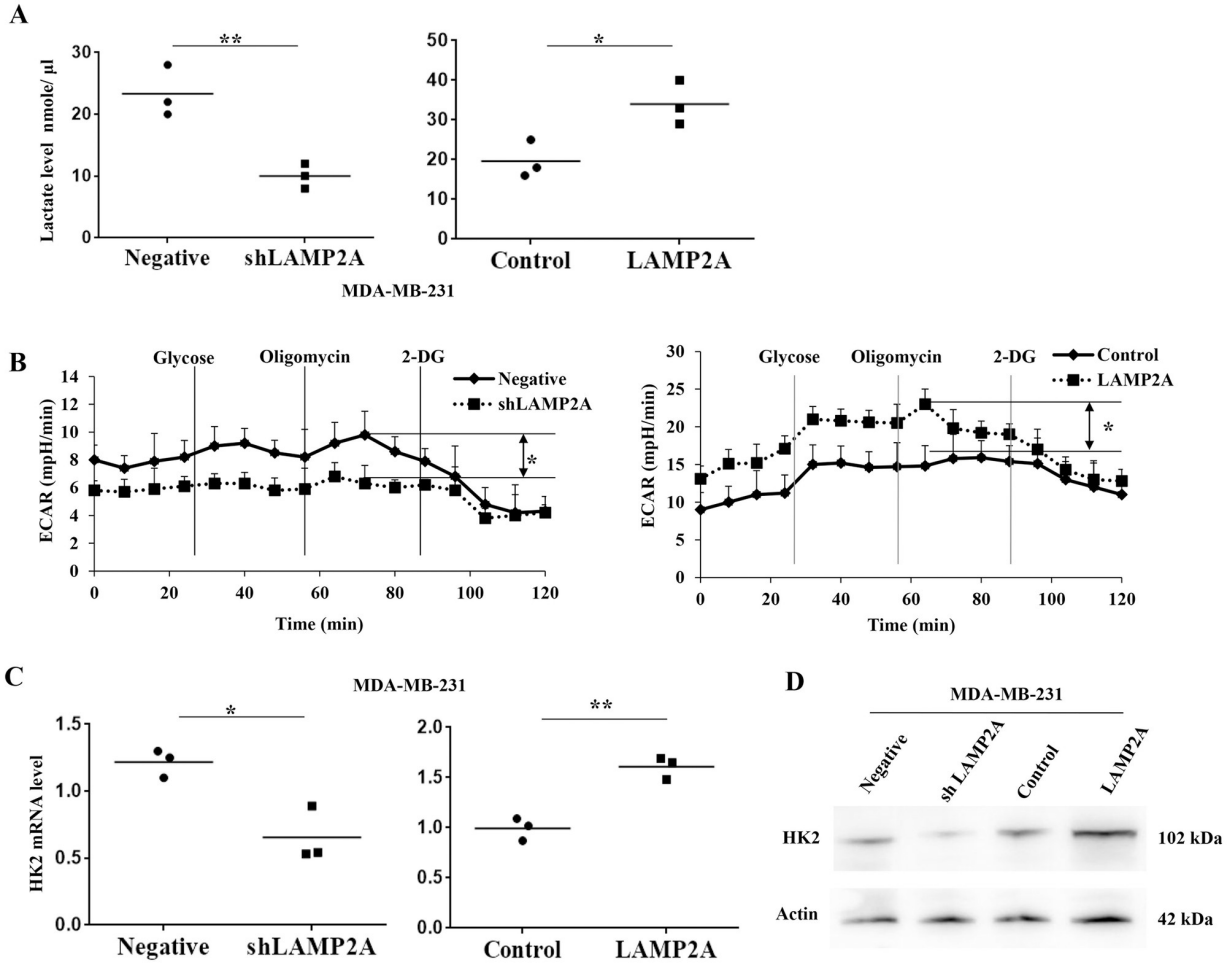
CMA promoted VEGFA expression through regulation of HK2-mediated aerobic glycolysis. (A) Lactate level was detected in TCM from MDA-MB-231 cells after LAMP2A knockdown or overexpression. (B) ECAR was detected in MDA-MB-231 cells with different LAMP2A expression, followed by injection of (vertical line) glucose (25 mM), oligomycin (1 μM), and 2-DG (50 mM). (C) HK2 mRNA expression level was detected by real-time PCR in MDA-MB-231 cells after LAMP2A knockdown or overexpression. (E) HK2 protein expression level was detected by Western blotting in MDA-MB-231 cells after LAMP2A knockdown or overexpression. All values are representative of three different experiments; *P < 0.05, **P < 0.01.

**Fig 7:**
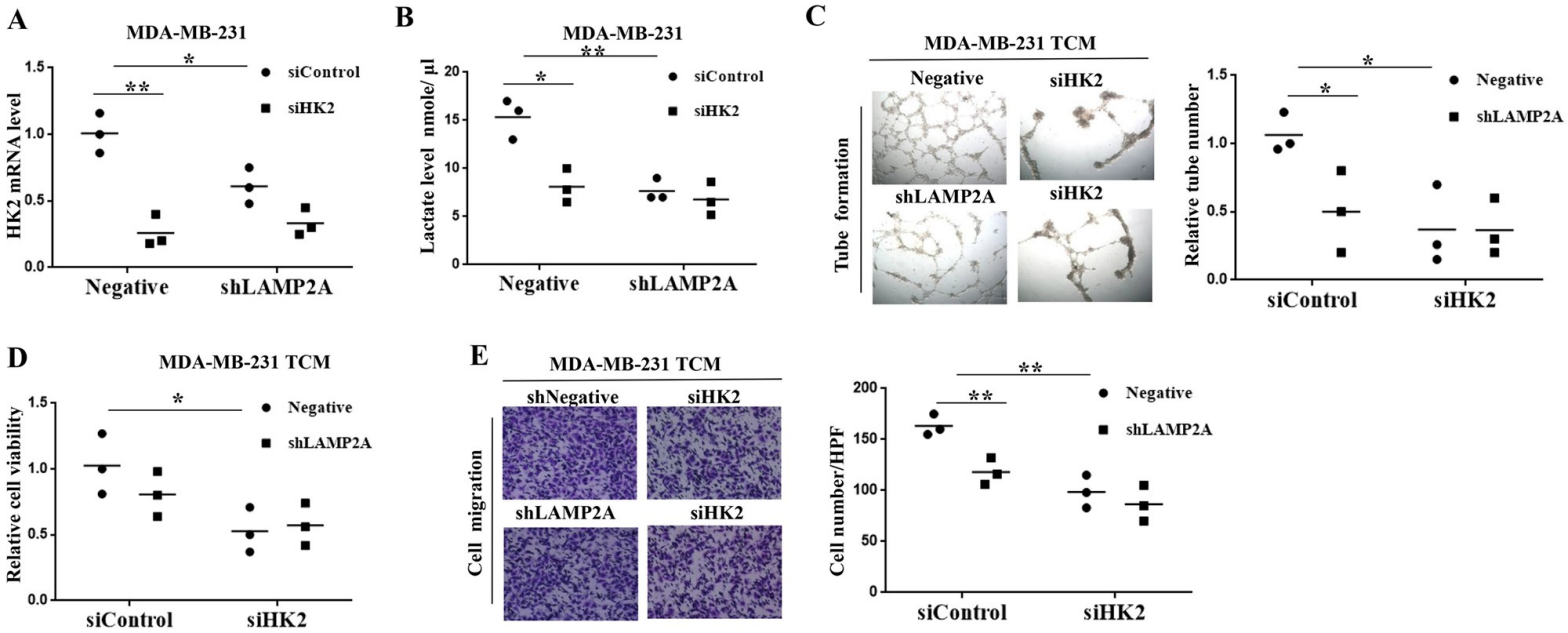
HK2 knockdown in breast cancer cells inhibited angiogenesis of HUVECs. (A) HK2 mRNA expression level was detected by real-time PCR in MDA-MB-231 cells after HK2 siRNA transfection. (B) Lactate level was detected in TCM from MDA-MB-231 cells after HK2 siRNA transfection. (C) TCM was collected from MDA-MB-231 cells after HK2 siRNA transfection, cocultured with HUVECs for 12 h, and then tubule numbers were counted via tube formation assay. (D) TCM collected from MDA-MB-231 cells after transfected with HK2 siRNA were cocultured with HUVECs for 48 h, and then cell viability of HUVECs was detected by MTT assay. (E) Cell migration of HUVECs was detected by Transwell migration assay after HK2 knockdown in MDA-MB-231 cells. All values are representative of three different experiments; *P < 0.05, **P < 0.01.

VEGFA expression level could be enhanced under hypoxia and elevated level of lactate [23], we then tested the effect of CMA activity on glycolysis pathway. We found that extracellular lactate level was lower in TCM from LAMP2A-knockdown breast cancer cells than those in negative control group, while higher lactate level was detected in TCM from LAMP2A-overexpressing breast cancer cells (Fig 6A). Then we used Seahorse system to investigate the extracellular acidification rate (ECAR) of breast cancer cells [24]. LAMP2A knockdown inhibited the basal ECAR compared with negative MDA-MB-231 cells, while LAMP2A overexpression upregulated the basal ECAR (Fig 6B). Since lactate is the final product of glycolysis, and HK2 was a key mediator in glycolysis pathway [25], we then determined HK2 levels in breast cancer cells. We found that HK2 mRNA expression level was downregulated after LAMP2A knockdown and increased after LAMP2A overexpression (Fig 6C). Then Western blotting further showed that LAMP2A knockdown led to decreased protein level of HK2 in breast cancer cells, while LAMP2A overexpression promoted the HK2 protein level in breast cancer cells (Fig 6D). To further evaluate the effect of aerobic glycolysis in breast cancer angiogenesis regulated by CMA, we used HK2 siRNA transfection and evaluated the capacity of angiogenesis. qRT-PCR analysis indicated that HK2 mRNA level was efficiently inhibited after HK2 siRNA transfection (Fig 7A), and lactate level was also downregulated after HK2 knockdown (Fig 7B). Then TCM was collected and cocultured with HUVECs, we found that knockdown of HK2 can significantly reduce the ability of CMA-mediated tube formation capacity of HUVECs (Fig 7C). Moreover, downregulation of HK2 can significantly reduce the ability of CMA-mediated cell growth and migration of HUVECs (Fig 7D and 7E). All these results showed that CMA promoted breast cancer angiogenesis through regulation of HK2-mediated aerobic glycolysis.

## Discussion

Angiogenesis plays an important role in cancer progression, and evidence has demonstrated its significance in aggressive malignancies [26]. Inhibiting angiogenesis pathway has long been a novel and effective target for the tumor treatment [27]. Angiogenesis is involved in a series of steps: detachment of endothelial cells, sprouting toward gradients, proliferation to form provisional tubes, finally, remodeling and forming a functional network [28]. In cancer, despite multiple factors and modes promoting endothelium-dependent angiogenesis [3], the potential role of CMA in breast cancer angiogenesis remains unknown. In this study, we first found that CMA could promote angiogenesis by HUVEC tube formation assay *in vitro*, while this phenomenon was not observed in human lung cancer cells [12], which may result from the high heterogeneity among different cancer types. Moreover, we also found that CMA could promote the proliferation and metastasis of HUVECs, which is important to the induction of angiogenesis. Our results suggest that CMA could promote breast cancer angiogenesis.

VEGFA mediates angiogenesis, the expansion of an existing vascular bed by sprouting of new blood vessels [28]. In our study, we found that VEGFA level was significantly decreased after LAMP2A knockdown and upregulated after LAMP2A overexpression *in vitro*, then we confirmed this result in tumor tissue. Evidence shows that lactate can promote angiogenesis through VEGF expression [29–31]. Lactate, the metabolic end-product of anaerobic glycolysis, is involved in carcinogenesis [7]. Cancer cells are characterized by increased aerobic glycolysis and excessive lactate formation. In our model, we also found that lactate level and baseline of glycolysis were significantly decreased after LAMP2A knockdown, and enhanced after LAMP2A overexpression. This was also consistent with other report [12]. Besides, among the key glycolytic enzymes, we found that HK2 was downregulated after LAMP2A knockdown and upregulated after LAMP2A overexpression, despite that HK2 has been characterized as CMA substrate [32]. The decreased level of HK2 did not result from an increase of degradation after LAMP2A knockdown, as qRT-PCR analysis showed significant decrease in the mRNA level of HK2. Moreover, we also found that knockdown of HK2 can result in less lactate generation and significantly reduce the ability of CMA-mediated tube formation capacity of HUVECs, which is consistent with other study [29].

## Conclusion

The present study provides a new link between CMA and angiogenesis, we show that CMA promotes VEGFA expression via regulation of HK2-mediated aerobic glycolysis, thus enhancing breast cancer angiogenesis. This helps us better understand the effects of CMA in tumorigenesis and metastasis, and suggests that CMA may be a promising target for breast cancer angiogenesis.

## Supporting information

S1 Fig Tumor growth of MDA-MB-436 cells with different LAMP2A expression in xenografts. 1 × 10^7^ shLAMP2A or Negative and LAMP2A overexpressing or Control MDA-MB-436 cells were subcutaneously injected into nude mice. The size of the tumors was monitored by the standard formula length × width × width × 0.5 (n = 5; *P < 0.05, **P < 0.01, shLAMP2A vs Negative, LAMP2A vs Control).
(DOC)

S1 File The ARRIVE essential 10. (DOC)

S1 Raw images (PDF)
