# Supplementary material for: Chaperone-mediated autophagy promotes breast cancer angiogenesis via regulation of aerobic glycolysis
Source: PLoS One. 2023 Mar 13;18(3):e0281577. doi: 10.1371/journal.pone.0281577 (PMC10010525; doi:10.1371/journal.pone.0281577)
Supplement: S1 Fig — 1 × 10^7^ shLAMP2A or Negative and LAMP2A overexpressing or Control MDA-MB-436 cells were subcutaneously injected into nude mice. The size of the tumors was monitored by the standard formula length × width × width × 0.5 (n = 5; *P < 0.05, **P < 0.01, shLAMP2A vs Negative, LAMP2A vs Control). (DOC) [file pone.0281577.s001.doc]

**Supplemental figures and figure legends:**

**
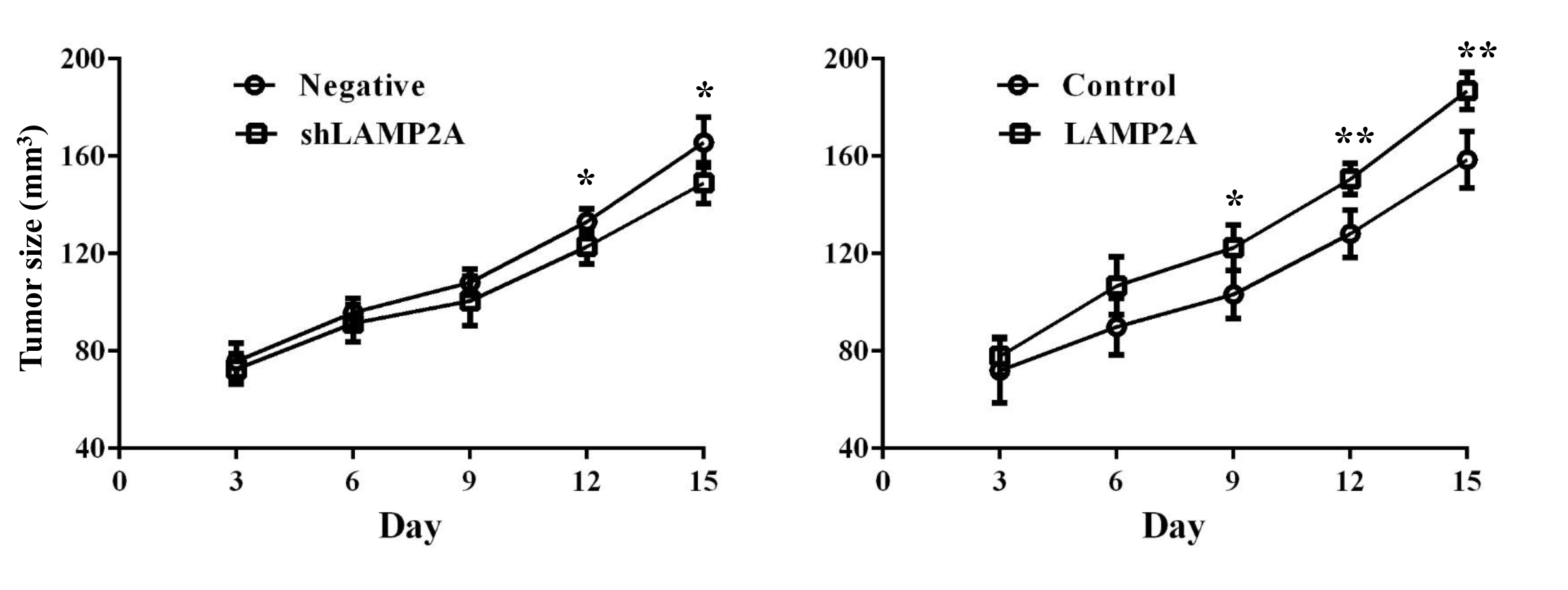
**

**Figure S1. Tumor growth of MDA-MB-436 cells with different LAMP2A expression in xenografts.**

1 × 10^7^ shLAMP2A or Negative and LAMP2A overexpressing or Control MDA-MB-436 cells were subcutaneously injected into nude mice. The size of the tumors was monitored by the standard formula length × width × width × 0.5 (n = 5; **P* < 0.05, ***P* < 0.01, shLAMP2A vs Negative, LAMP2A vs Control).
