# Supplementary material for: Chaperone-mediated autophagy promotes breast cancer angiogenesis via regulation of aerobic glycolysis
Source: PLoS One. 2023 Mar 13;18(3):e0281577. doi: 10.1371/journal.pone.0281577 (PMC10010525; doi:10.1371/journal.pone.0281577)
Supplement: S1 File — (DOC) [file pone.0281577.s002.doc]

**The ARRIVE Essential 10**

**Study design**

1 For each experiment, provide brief details of study design including:

a. The groups being compared, including control groups. If no control group has been used, the

rationale should be stated.

**Response:** Yes, the groups being compared, including control groups.

b. The experimental unit (e.g. a single animal, litter, or cage of animals).

**Response:** The experimental unit is a single animal.

**Sample size**

2

a. Specify the exact number of experimental units allocated to each group, and the total number

in each experiment. Also indicate the total number of animals used.

**Response:** We have four groups, and we used 20 mice in total. Each group had five mice. All mice were successfully transplanted with tumors.

b. Explain how the sample size was decided. Provide details of any a priori sample size

calculation, if done.

**Response:** The sample size was decided by the experience of our previous study (Downregulation of ATG5-dependent macroautophagy by chaperone-mediated autophagy promotes breast cancer cell metastasis. Sci Rep. 2017;7(1):4759. doi:10.1038/s41598-017-04994-x).

**Inclusion and exclusion criteria**

3

a. Describe any criteria used for including and excluding animals (or experimental units) during

the experiment, and data points during the analysis. Specify if these criteria were established

a priori. If no criteria were set, state this explicitly.

**Response:** Mice that failed to take the tumor or died during the trial were excluded, a criteria that was established before the experiment began.

b. For each experimental group, report any animals, experimental units or data points not

included in the analysis and explain why. If there were no exclusions, state so.

**Response:** We have four groups, and we used 20 mice in total. Each group had five mice. All mice were successfully transplanted with tumors. No mice is excluded in the analysis.

c. For each analysis, report the exact value of n in each experimental group.

**Response:** We have four groups, and we used 20 mice in total. Each group had five mice (n=5).

**Randomisation**

4

a. State whether randomisation was used to allocate experimental units to control and

treatment groups. If done, provide the method used to generate the randomisation sequence. **Response:** Yes, randomisation was used to allocate experimental units to control and

treatment groups. We use a random number table for complete randomization of groups.

b. Describe the strategy used to minimise potential confounders such as the order of

treatments and measurements, or animal/cage location. If confounders were not controlled,

state this explicitly.

**Response:** The mice in each group were also treated or measured in a random catch manner.

**Blinding**

5

Describe who was aware of the group allocation at the different stages of the experiment (during

the allocation, the conduct of the experiment, the outcome assessment, and the data analysis). **Response:** Different operators are responsible for different stages of the experiment, and no information is shared among the operators.

**Outcome measures**

6

a. Clearly define all outcome measures assessed (e.g. cell death, molecular markers, or

behavioural changes).

**Response:** Tumor size and VEGFA level in tumor tissue (Western blotting) of the mice were assessed.

b. For hypothesis-testing studies, specify the primary outcome measure, i.e. the outcome

measure that was used to determine the sample size.

Response: VEGFA level was the primary outcome measure.

**Statistical methods**

7

a. Provide details of the statistical methods used for each analysis, including software used. **Response:** The protein expression levels can be directly perceived through Western blot, so descriptive statistics analysis was used.

b. Describe any methods used to assess whether the data met the assumptions of the

statistical approach, and what was done if the assumptions were not met.

**Response:** None.

**Experimental animals**

8

a. Provide species-appropriate details of the animals used, including species, strain and

substrain, sex, age or developmental stage, and, if relevant, weight.

**Response:** 4-6-week-old female BALB/c-nu/nu nude mice were used in the experiment.

b. Provide further relevant information on the provenance of animals, health/immune status,

genetic modification status, genotype, and any previous procedures.

**Response:** The provenance of animals: The Experimental Animal Center, Army Medical University, Chongqing, China; Health/immune status: Health/Immune system deficiencygenetic;

Modification status: None; Genotype: Ordinary Nude Mouse.

**Experimental procedures**

9

For each experimental group, including controls, describe the procedures in enough detail to

allow others to replicate them, including:

1. What was done, how it was done and what was used.

**Response:** 20 four-six-week-old female BALB/c nude mice in total were randomly divided into four groups, and each group had five mice (n=5). Mice were held at the Experimental Animal Center of Army Medical University for one week before injection, and the health status and well-being of the mice were checked twice a day by the animal care staff. Mice were injected with MDA-MB-436 cells (in 200 µl of serum-free RPMI-1640 medium at 1 × 10^7^ cells/mouse) after LAMP2A knockdown or overexpression at the right flank. When tumor volumes of negative and control groups reached about 150 mm^3^ (tumor size=length × width × width × 0.5), animals were sacrificed by CO_2_ euthanasia, and all efforts were made to minimize suffering, then the subcutaneous tumors were collected and used for Western blot.

1. When and how often.

**Response:** When tumor volumes of negative and control groups reached about 150 mm^3^ (tumor size=length × width × width × 0.5), animals were sacrificed by CO_2_euthanasia, and all efforts were made to minimize suffering, then the subcutaneous tumors were collected.

1. Where (including detail of any acclimatisation periods).

**Response:** All the animal experiments were carried out in SPF animal laboratory (The Experimental Animal Center of Army Medical University).

1. Why (provide rationale for procedures).

**Response:** All animal studies were approved by the Ethics Committee of Army Medical University and all the procedures were performed according to the regulations made by the Experimental Animal Center of Army Medical University.

**Results**

10

For each experiment conducted, including independent replications, report:

a. Summary/descriptive statistics for each experimental group, with a measure of variability

where applicable (e.g. mean and SD, or median and range).

**Response:** The protein expression levels can be directly perceived through Western blot, so descriptive statistics analysis was used.

b. If applicable, the effect size with a confidence interval.

**Response:** None.

**The Recommended Set**

**Abstract**

11

Provide an accurate summary of the research objectives, animal species, strain and sex,

key methods, principal findings, and study conclusions.

**Response:** 20 four-six-week-old female BALB/c nude mice in total were randomly divided into four groups, and each group had five mice (n=5). Mice were held at the Experimental Animal Center of Army Medical University for one week before injection, and the health status and well-being of the mice were checked twice a day by the animal care staff. Mice were injected with MDA-MB-436 cells (in 200 µl of serum-free RPMI-1640 medium at 1 × 10^7^ cells/mouse) after LAMP2A knockdown or overexpression at the right flank. When tumor volumes of negative and control groups reached about 150 mm^3^ (tumor size=length × width × width × 0.5), animals were sacrificed by CO_2_ euthanasia, and all efforts were made to minimize suffering, then the subcutaneous tumors were collected and used for Western blot. The results indicated that LAMP2A knockdown significantly inhibited VEGFA expression in tumors from the shLAMP2A MDA-MB-436 implanted mice, compared with tumors from the negative MDA-MB-436 implanted mice, while LAMP2A overexpression significantly promoted VEGFA expression in tumors from the LAMP2A MDA-MB-436 implanted mice, compared with tumors from the control MDA-MB-436 implanted mice

**Background**

12

a. Include sufficient scientific background to understand the rationale and context for the

study, and explain the experimental approach.

**Response:** Subcutaneous tumor model of xenograft in nude mice is a classic model, which can provide us an *in vivo* simulation environment for tumor research.

b. Explain how the animal species and model used address the scientific objectives and,

where appropriate, the relevance to human biology.

**Response:** Because of the immune deficiency in nude mice, tissue transplants from xenografts are not rejected under certain conditions. Therefore, it can be used as the recipient of human malignant tumor transplantation. Human tumor transplanted in immunodeficient animals can maintain its biological characteristics, and is suitable for the study of drug sensitivity of human tumor.

**Objectives**

13

Clearly describe the research question, research objectives and, where appropriate,

specific hypotheses being tested.

**Response:** To detect the expression level of VEGFA after manipulating CMA activity *in vivo*, we conducted the xenograft studies.

**Ethical statement**

14

Provide the name of the ethical review committee or equivalent that has approved the

use of animals in this study, and any relevant licence or protocol numbers (if applicable). If

ethical approval was not sought or granted, provide a justification.

**Response:** All animal studies were approved by the Ethics Committee of Army Medical University and all the procedures were performed according to the regulations made by the Experimental Animal Center of Army Medical University.

**Housing and husbandry**

15

Provide details of housing and husbandry conditions, including any environmental

enrichment.

**Response:** The animals live in the SPF laboratory animal room and are cared for by professional staff.

**Animal care and monitoring**

16

a. Describe any interventions or steps taken in the experimental protocols to reduce pain,

suffering and distress.

**Response:** Animals were sacrificed by CO_2_ euthanasia, and all efforts were made to minimize suffering

b. Report any expected or unexpected adverse events.

**Response:** None.

c. Describe the humane endpoints established for the study, the signs that were

monitored and the frequency of monitoring. If the study did not have humane endpoints,

state this.

**Response:** The mice were euthanized with CO_2_. The euthanasia device, which is airtight and has good transparency, is convenient for observing whether the animal is dead during operation, is carried out in a well-ventilated environment. Before putting the mouse into the euthanasia device, a certain amount of carbon dioxide is introduced, and then the mouse is put into it, so that the mouse can enter the anesthesia state quickly and reduce fear and pain. After the animal dies, continue to infuse carbon dioxide for 2 to 3 minutes.

**Interpretation/ scientific implications**

17

a. Interpret the results, taking into account the study objectives and hypotheses, current

theory and other relevant studies in the literature.

**Response:** These contents have been elaborated in **Discussion**.

b. Comment on the study limitations including potential sources of bias, limitations of the

animal model, and imprecision associated with the results.

**Response:** None.

**Generalisability/ translation**

18

Comment on whether, and how, the findings of this study are likely to generalise to other

species or experimental conditions, including any relevance to human biology (where

appropriate).

**Response:** The cell line we used in the study was derived from human breast cancer tissues and the animals were also mammals, so the results are applicable to species with similar biological characteristics.

**Protocol registration**

19

Provide a statement indicating whether a protocol (including the research question, key

design features, and analysis plan) was prepared before the study, and if and where this

protocol was registered.

**Response:** The research question, key design features, and analysis plan were prepared and registered before the study in the Ethics Committee of Army Medical University.

**Data access**

20

Provide a statement describing if and where study data are available.

**Response:** All data have been presented in the manuscript and Supporting Information files.

**Declaration of interests**

21

a. Declare any potential conflicts of interest, including financial and non-financial. If none

exist, this should be stated.

**Response:** All authors have no competing interests.

b. List all funding sources (including grant identifier) and the role of the funder(s) in the

design, analysis and reporting of the study.

**Response:** This work was supported by National Natural Science Foundation of China (82072946) and Natural Science Foundation of Tibet (XZ202101ZR0109G). The funders had no role in study design, data collection and analysis, decision to publish, or preparation of the manuscript.

.
