# Supplementary material for: Chaperone-mediated autophagy promotes breast cancer angiogenesis via regulation of aerobic glycolysis
Source: PLoS One. 2023 Mar 13;18(3):e0281577. doi: 10.1371/journal.pone.0281577 (PMC10010525; doi:10.1371/journal.pone.0281577)

# Raw band for Figure 1A

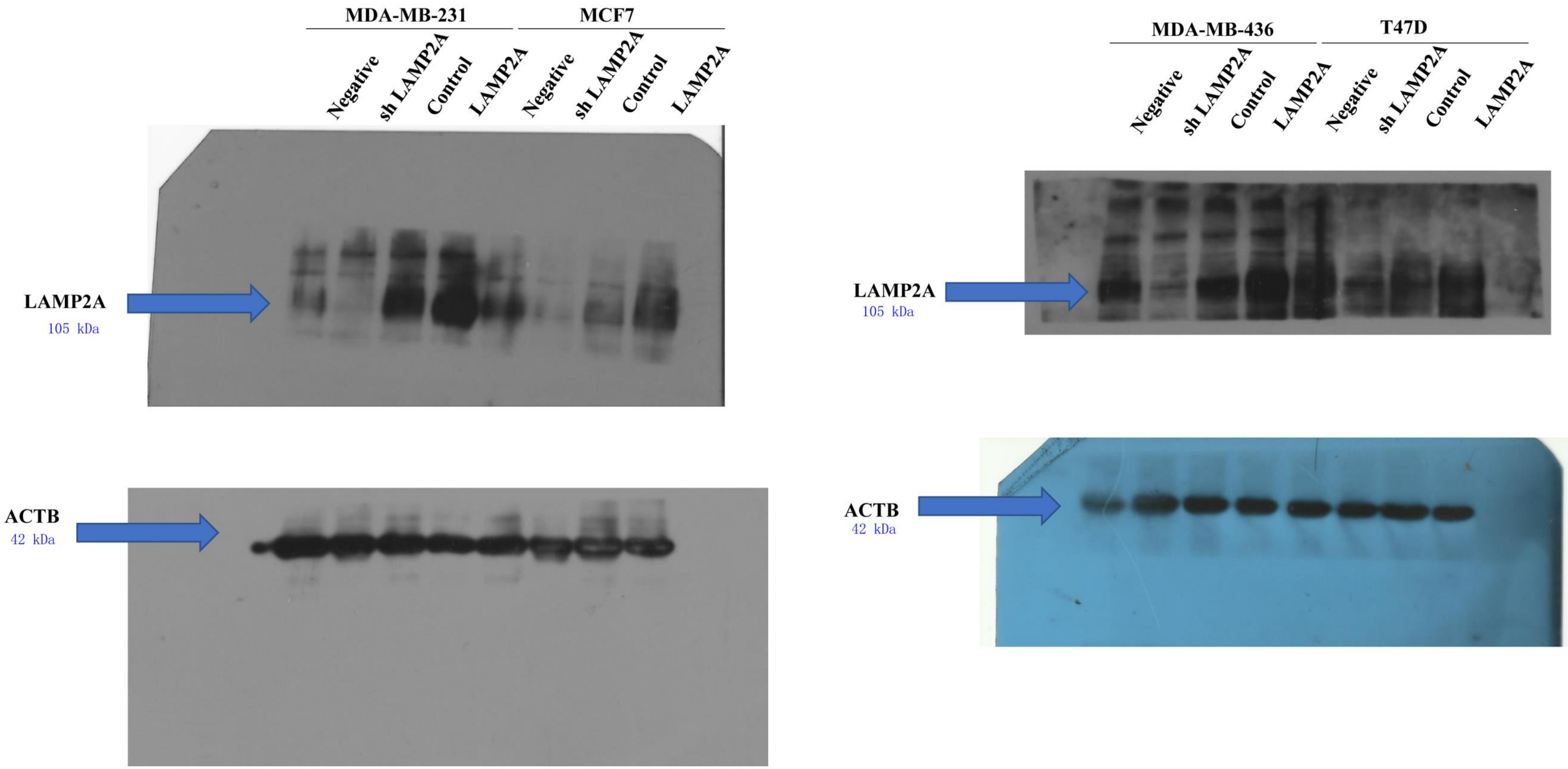

# Raw band for Figure 1B

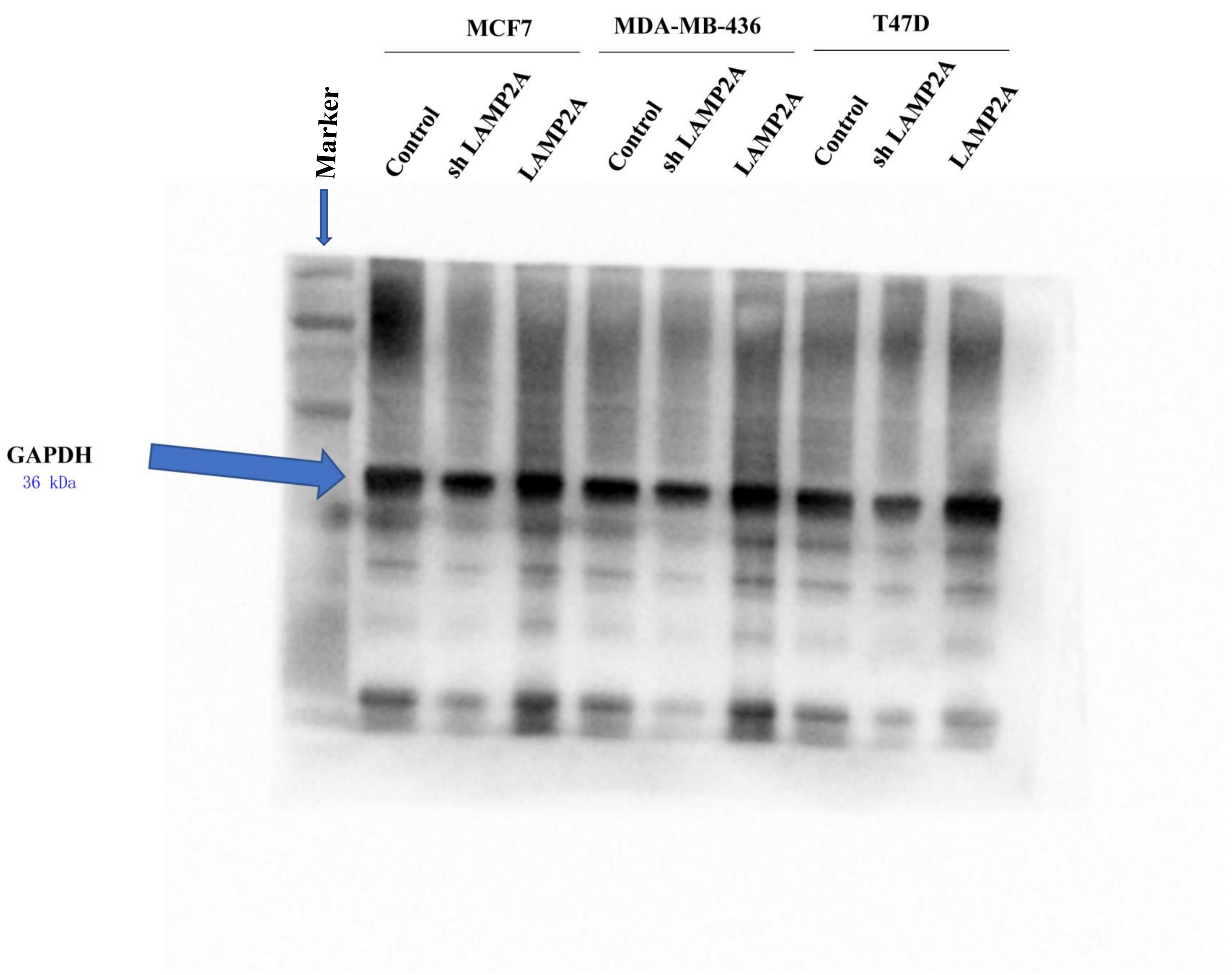

# Raw band for Figure 5B

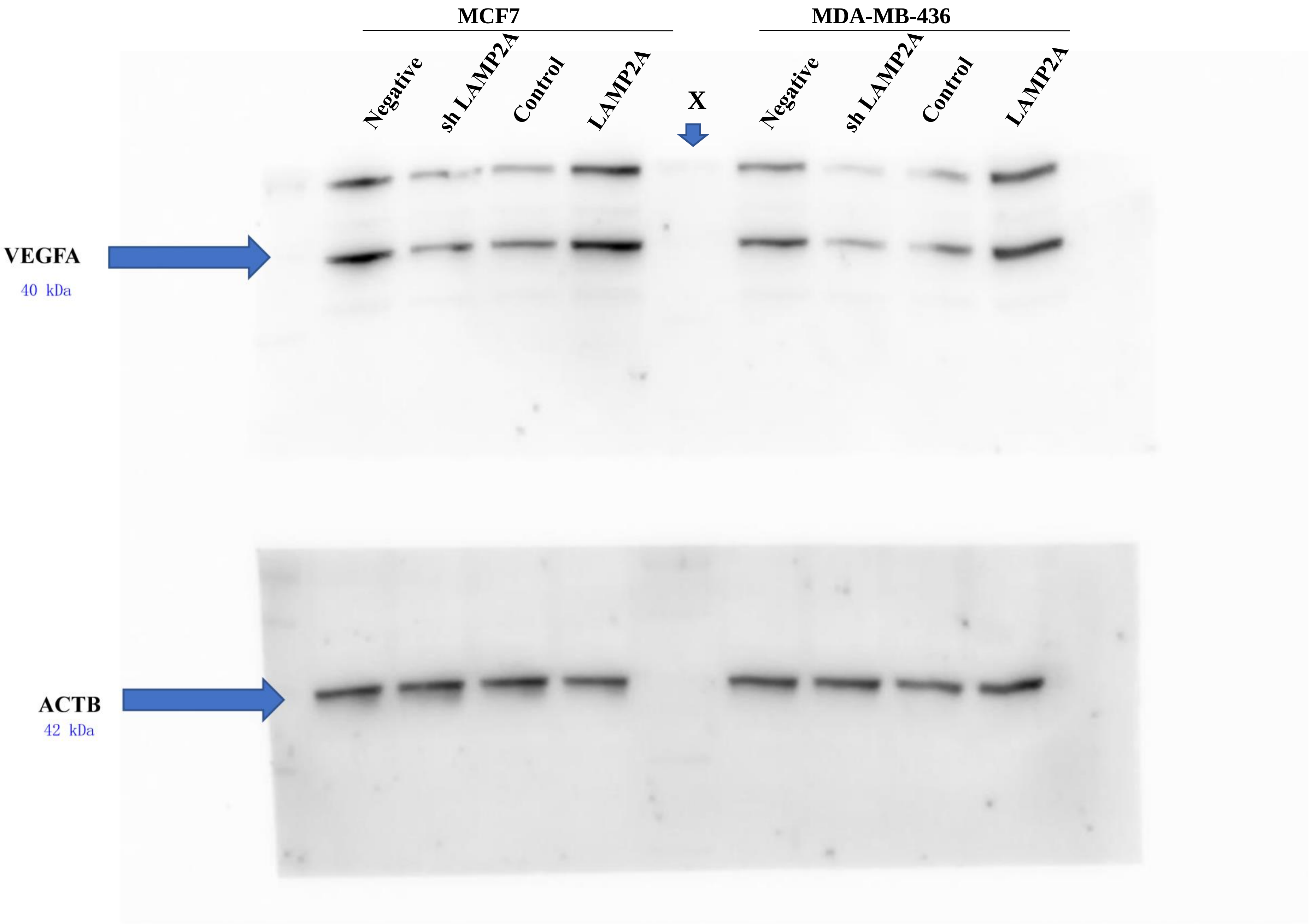

# Raw band for Figure 5D

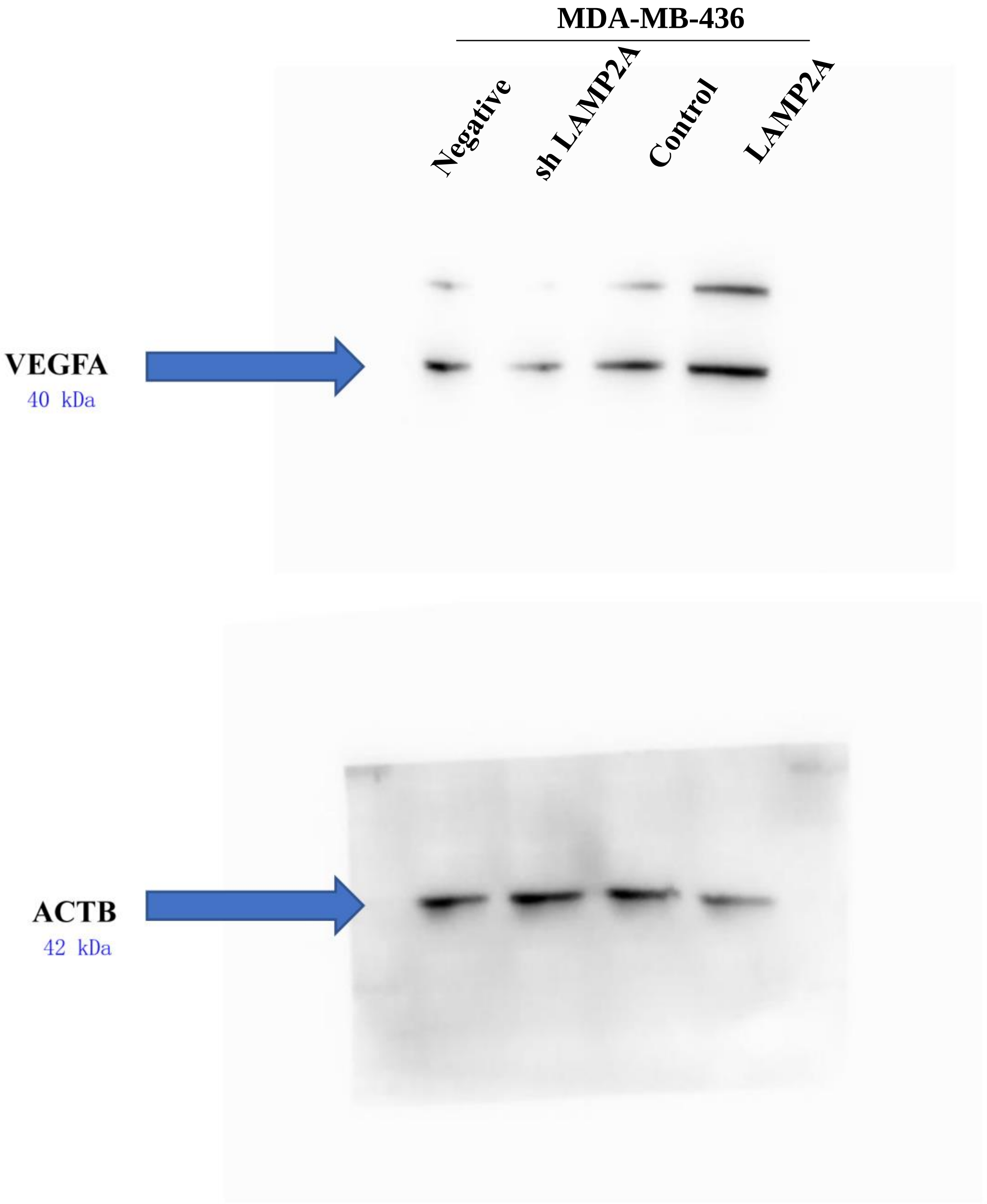

# Raw band for Figure 6D

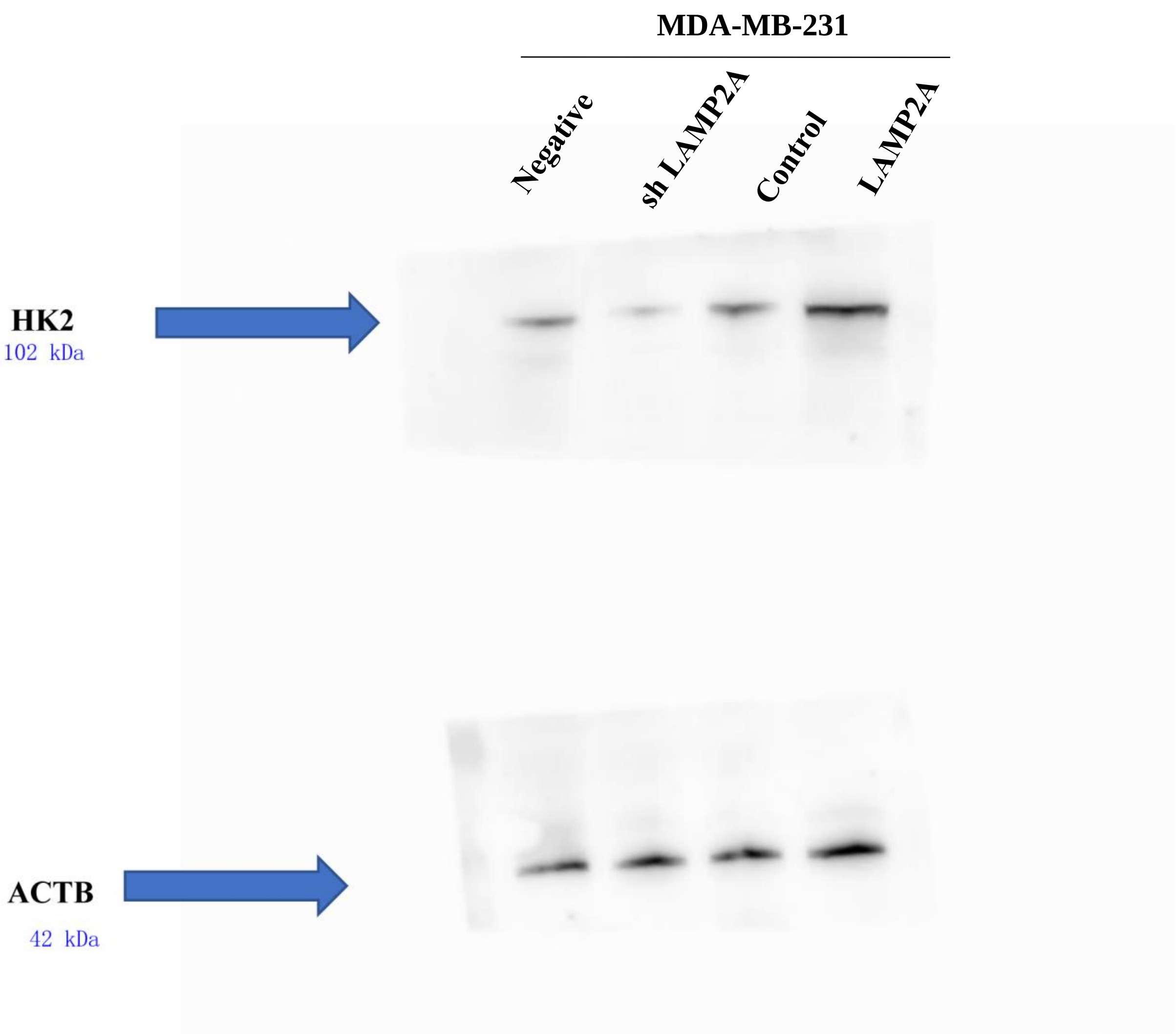

Supplement: S1 Raw images — (PDF) [file pone.0281577.s003.pdf]
